# Nitrile as Activating Group in the Asymmetric Bioreduction of β-Cyanoacrylic Acids Catalyzed by Ene-Reductases

**DOI:** 10.1002/adsc.201301055

**Published:** 2014-04-09

**Authors:** Christoph K Winkler, Dorina Clay, Nikolaus G Turrini, Horst Lechner, Wolfgang Kroutil, Simon Davies, Sebastien Debarge, Pat O'Neill, Jeremy Steflik, Mike Karmilowicz, John W Wong, Kurt Faber

**Affiliations:** a Department of Chemistry, Organic & Bioorganic Chemistry, University of Graz Heinrichstrasse 28, A-8010 Graz, Austria, Fax: (+43)-316-380-9840; phone: (+43)-316-380-5332; e-mail: kurt.faber@uni-graz.at; b Pfizer Global Supply, Process Development Centre Loughbeg, County Cork, Ireland; c Pfizer Worldwide R&D, Chemical R&D Eastern Point Rd, Groton, CT 06340, USA

**Keywords:** biocatalysis, C=C reduction, cyanoacrylates, ene-reductases, pregabalin

## Abstract

Asymmetric bioreduction of an (*E*)-β-cyano-2,4-dienoic acid derivative by ene-reductases allowed a shortened access to a precursor of pregabalin [(*S*)-3-(aminomethyl)-5-methylhexanoic acid] possessing the desired configuration in up to 94% conversion and >99% *ee*. Deuterium labelling studies showed that the nitrile moiety was the preferred activating/anchor group in the active site of the enzyme over the carboxylic acid or the corresponding methyl ester.

The stereoselective reduction of C=C-bonds represents a powerful tool for asymmetric synthesis, which allows the introduction of up to two stereogenic centers in a single step. While transition metal and organocatalytic methods are already widely applied in asymmetric C=C-bond reduction,[1,2] the biocatalytic equivalent based on ene-reductases was developed more recently.[3] These flavin-dependent members of the *old yellow enzyme* (OYE) family catalyze the stereoselective reduction of alkenes, which are activated by an electron-withdrawing group, such as carbonyl (encompassing aldehydes, ketones, carboxylic acids, esters, lactones, nitriles and cyclic imides) or nitro groups.[4,5] We recently demonstrated the applicability of ene-reductases in the chemoenzymatic asymmetric synthesis of non-racemic precursors for several GABA-analogues, such as pregabalin and brivaracetam, via stereoselective reduction of a library of *β*- cyanoacrylate esters.[6] The double activation of the C=C-bond by the carboxylic ester and nitrile moieties, which are both known as weak electron-withdrawing groups[7–9] renders these substrates ideal candidates for ene-reductases, which translated to high conversions and perfect enantioselectivities. However, the use of esters as substrates requires an additional hydrolysis step in the downstream synthesis of the γ-aminocarboxylic acid pharmacophores. In order to avoid this we anticipated a shortcut by using the corresponding β-cyano acids instead of the corresponding esters, despite the disadvantage of their lower degree of C=C-bond activation in comparison to carboxylic acids.[7] The latter strategy would furnish the final products in a single step by catalytic hydrogenation (Scheme 1).

**Scheme 1:**
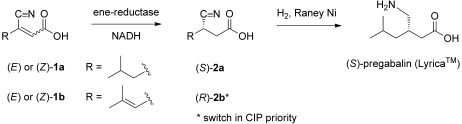
Asymmetric bioreduction of β-cyanoacrylic acids and hydrogenation to pregabalin [(*S*)-3-(aminomethyl)-5-methylhexanoic acid].

Cyano acids (*E*)-**1a** and (*Z*)-**1a** were prepared by hydrolysis of the corresponding ethyl esters, (*E*)- and (*Z*)-ethyl 5-methyl-3-cyanohex-2-enoates, which were obtained as previously described.[6] (*E*)-**1b** was made by a one-pot Knoevenagel condensation of isobutyraldehyde and cyanoacetic acid to give 2-cyano-4-methylpent-2-enoic acid [(*E/Z*)-**3**], followed by a decarboxylative Knoevenagel reaction with glyoxylic acid to give (*E*)-**1b** as the desired product (Scheme 2). Although (*Z*)-**1b** was also formed in this process, it was readily converted to 2-(2-methylprop-1-enyl)maleic acid (**4**) under the acidic conditions of the reaction. Alternatively, (*Z*)-**1b** was successfully prepared by a DABCO-catalyzed addition reaction of 2-cyano-4-methylpent-2-enoic acid (**3**) and glyoxylic acid to give 3-cyano-2-hydroxy-5-methylhex-4-enoic acid (**5**) as a mixture of diastereomers. Dehydration of this material was readily achieved by treatment with K_2_CO_3_ in MeOH to afford (*Z*)-**1b** as the potassium salt, which was stable and did not undergo decomposition to **4**, as was observed for the free acid.

**Scheme 2:**
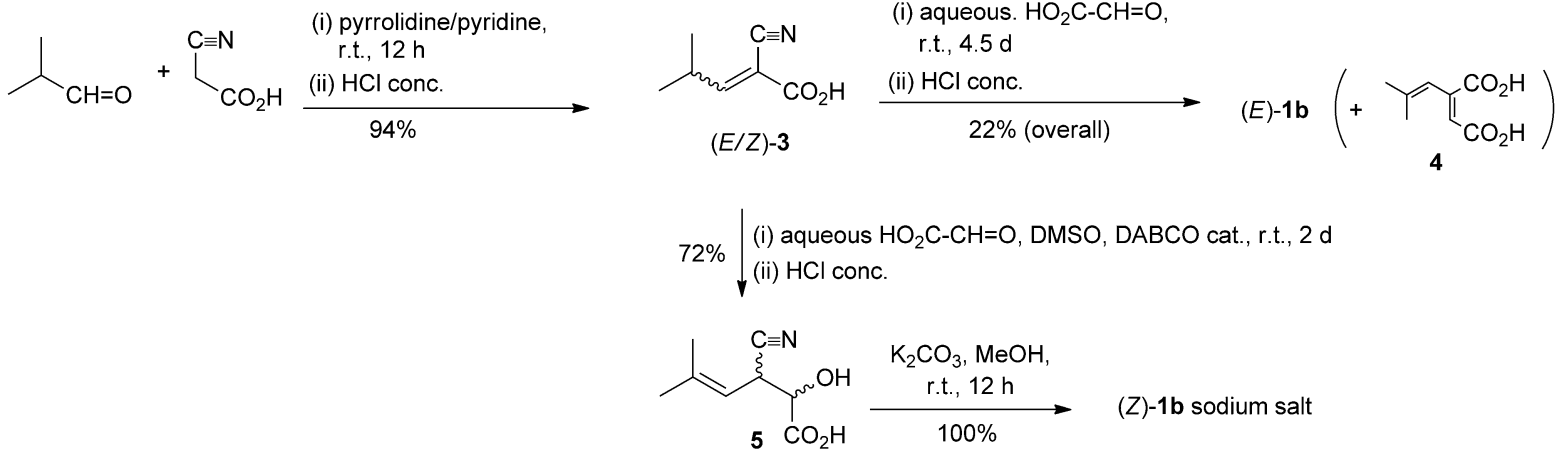
Synthesis of β-cyanoacrylic acids

Both stereochemically pure (*E*)*-* and (*Z*)-forms of substrates **1a** and **1b** (handled as sodium/potassium salts) were tested with a set of eleven purified ene-reductases and three single-point mutants of 12-oxophytodienoic acid reductase (OPR1).[6] As expected from our previous comparative studies on α,β-unsaturated acids and esters,[7] the substrate acceptance was generally lower compared to the corresponding esters (Table 1).[6] To our disappointment, the more flexible substrates (*E*)- and (*Z*)-**1a** containing a single α,β-C=C-bond were unsuitable: The (*Z*)-isomer was not converted by any of the tested enzymes (0% conversion) and (*E*)-**1a** gave conversions at the limit of detectability (≤3%, OPR1-wt, mutant OPR1-I287H). Surprisingly, the picture changed with the more rigid doubly unsaturated acids (*E*)- and (*Z*)-**1b**. Whereas (*Z*)-**1b** still was a bad substrate for any of the tested enzymes, (*E*)-**1b** could be reduced with high conversion and perfect stereoselectivities to the desired (*R*)-enantiomer of **2b** (note the switch in CIP priorities between **2a** and **2b**). Among the tested enzymes, OPR1-wt and three OPR1-mutants were clearly best. Consequently, substrate (*E*)-**1b** was used to optimize pH and buffer type, which revealed Tris-HCl buffer at pH 7.5 (100 mM) as the optimum conditions (see the Supporting Information). Preparative-scale bioreduction of (*E*)-**1b** with OPR1-wt afforded a quantitative yield of the desired (*R*)-**2b** (1.15 g) with perfect enantioselectivity demonstrating the potential utility of this transformation for large-scale synthetic applications.

**Table 1:** Asymmetric bioreduction of (*E*)-1a and (*E*)-1b

Substrate	Product	Enzyme^[a]^	Conversion [%]	*ee* [%]
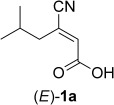	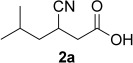	OPR1-wt	≤3	n.d.^[b]^
		OPR1-I287H	≤3	n.d.^[b]^
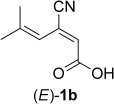	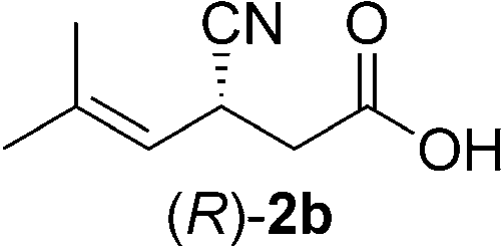	OYE3	≤3	>99
		*Gk*OYE	≤3	>99
		OYE2	3	>99
		EBP1	6	>99
		OPR1-I287H	68	>99
		OPR1-H245D	74	>99
		OPR1-C20Y	91	>99
		OPR1-wt	96	>99^[c]^

^[a]^ 12-Oxophytodienoic acid reductase isoenzymes OPR1 and OPR3 (*Lycopersicon esculentum*), OPR1 variants: I287H, H245D, C20Y; OYE homologue YqjM (*Bacillus subtilis*), OYE1 (*Saccharomyces pastorianus*), OYE2 and OYE3 (*Saccharomyces cerevisiae*), nicotinamide-dependent cyclohexenone reductase NCR (*Zymomonas mobilis*), Xenobiotic reductase XenA (*Pseudomonas putida*), estrogen binding protein EBP1 (*Candida albicans*), *Gk*OYE (*Geobacillus kaustophilus* DSM 7263) and CrS (*Thermus scotoductus* SA-01); the following enzymes were inactive (data not shown): OYE1, OPR3, YqjM, NCR, XenA and CrS.

^[b]^ n.d.=not determined.

^[c]^ Preparative-scale reduction (1.15 g).

The absolute configuration of products (*S*)-**2a** and (*R*)-**2b** was determined *via* co-injection of the biotransformation products after derivatization to the corresponding methyl esters with independently synthesized reference samples on chiral GC.

In order to rationalize the unexpected acceptance of only a single substrate out of four closely related isomeric derivatives, we investigated the mode of substrate positioning with respect to the nature of the activating group, that is, carboxylic acid *versus* nitrile, both of which are known to act as moderate activating groups.[7,8,10] The mechanism of OYEs is well understood:[11] The substrate is oriented such that (i) the electron-withdrawing group is tightly bound onto a pair of H-donor residues in the back of the active site (either two His or His/Asn),[12] which (ii) positions the Cβ atom of the α,β-C=C-bond to be reduced close to N-5 of the flavin cofactor bearing the hydride. (iii) The corresponding proton required for reduction is delivered from the opposite side onto Cα by an acidic residue (usually Tyr, occasionally Cys, the proton is ultimately derived from water) resulting in a general *trans*-addition.[4,5] When the bioreduction is conducted in D_2_O as solvent using NADH as hydride donor, Cα will be labelled, while Cβ will not (Scheme 3).[13–15]

**Scheme 3:**
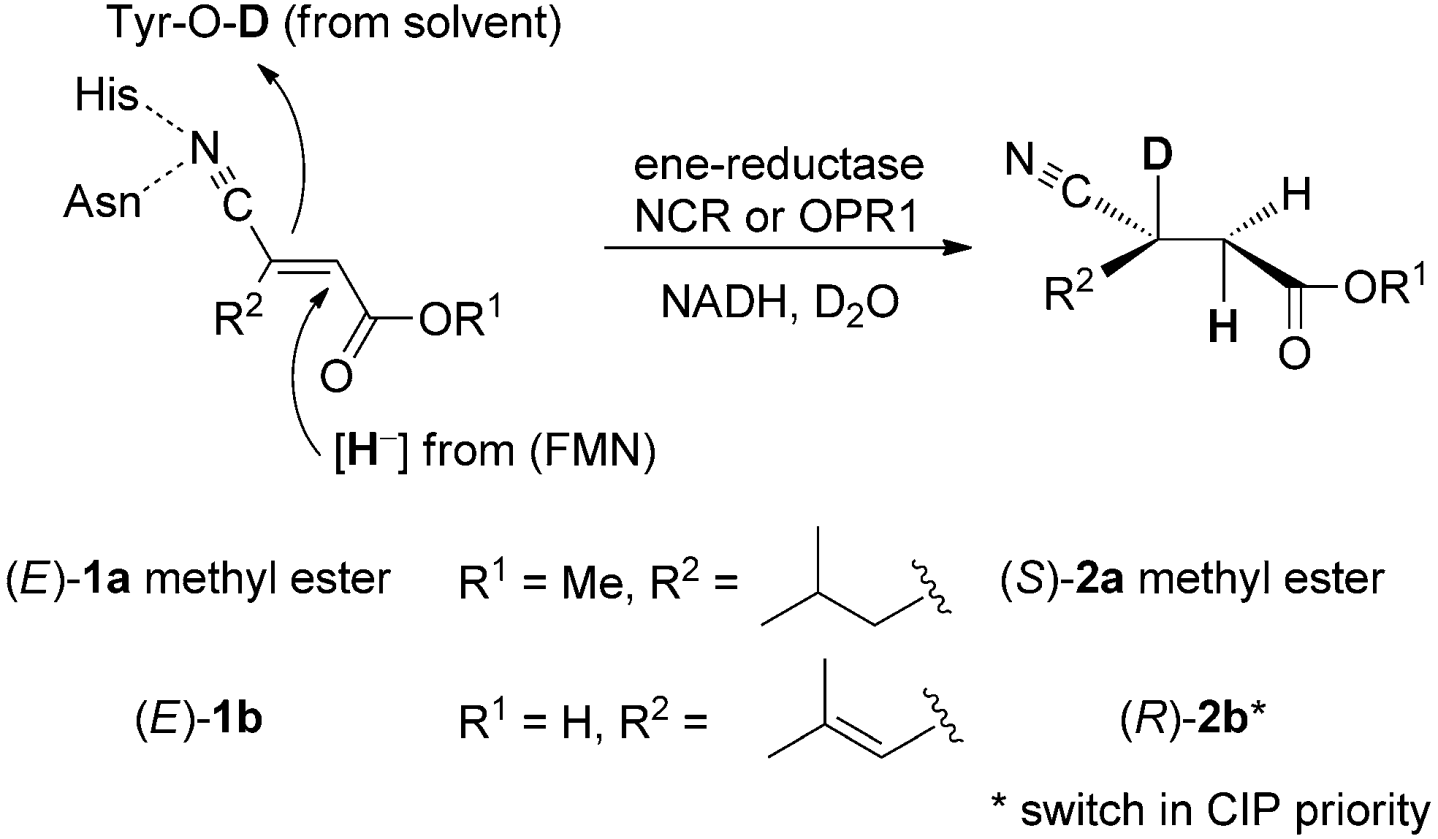
Deuterium labelling during bioreduction of (*E*)-1b using OPR1-wt and (*E*)-1a methyl ester using NCR and NADH in D_2_O.

NMR analysis of product **2b** obtained from the bioreduction of (*E*)-**1b** using OPR1-wt and NADH in D_2_O revealed exclusive deuterium-labelling at Cα of the nitrile group (≥ 97%, indicated by the disappearance of the H signal), which revealed the C≡N rather than the carboxylate moiety being bound as activating group. Since carboxylic acids, esters and nitriles are generally regarded as borderline substrates due to their limited degree of activation,[5c] we investigated also the corresponding methyl ester of (*E*)-**1a**,[6] by anticipating that the enhanced carbonyl activity of the ester would convert it to the preferred activating group over the nitrile *via* a flipped binding mode of the substrate.[13,16] Much to our surprise, the deuteration pattern remained unchanged, which proves that the nitrile remained the dominant activating group.

In order to rationalize the preference of the nitrile as activating group and the surprisingly narrow substrate spectrum, docking of (*E*)-**1b** into OPR1 (PDB-code: 3HGR)[18] was performed (Figure 1). The resulting structure with the highest docking score clearly supports the deuterium-labelling results: the nitrile moiety of the substrate is hydrogen-bonded by His187 and His190 within a distance of 2.9 and 2.0 Å, respectively. The binding mode correctly predicts the formation of (*R*)-**2b**, by placing Cβ in a favourable position for hydride transfer from FMN-N-5 across a distance of 3.9 Å. Additionally, the prochiral carbon (Cα) is in close proximity to the hydroxy group of Tyr192 (2.6 Å) which acts as proton donor. In contrast, other binding modes of (*E*)-**1b** gave only arrangements in which the C=C-bond was oriented in an unproductive fashion[16] with distances of >4 Å to FMN-N-5 and Tyr192, while the nitrile invariably remained bound onto His187 and His190. Attempts to force substrate (*E*)-**1b** to adopt a flipped binding mode through hydrogen bonding of its carboxylate group (instead of its nitrile) to His187 and His190 failed and did not produce any productive binding mode.

**Figure 1:**
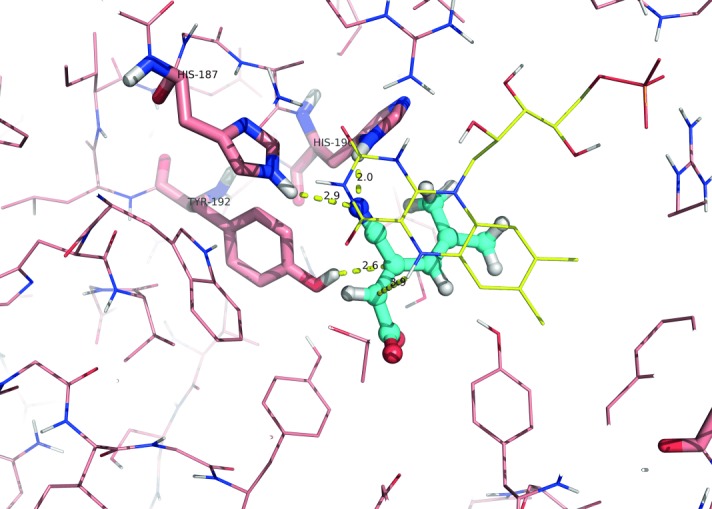
Docking of (*E*)-1b within the active site of OPR1 (PDB code: 3HGR): FMN (yellow), activating His187/His 190 and proton donating Tyr192 (purple), substrate (cyan).

Asymmetric bioreduction of a β-cyanoacrylic acid derivative by ene-reductases allowed a shortened access to a pregabalin precursor possessing the desired configuration in up to quantitative conversion and >99% *ee* The presence of an additional C=C-bond in the γ,δ-position proved to be vital for substrate acceptance and deuterium labelling studies showed that the nitrile moiety was the preferred activating/anchor group in the active site of the enzyme over the carboxylic acid or the corresponding methyl ester, which was supported by molecular modelling studies.

## Experimental Section

### General

GC analyses were carried out on an Agilent 7890A using a Hydrodex-β-TBDAc capillary column (25 m×0.25 mm id) using H_2_ as carrier gas or on an Agilent 6890N using a Chiraldex G-TA column (30 m×0.25 mm id) using He as carrier gas. NMR spectra were obtained on a Bruker 400 or 300 MHz NMR spectrometer. Chemical shifts are reported relative to TMS (*δ*=0.00) and coupling constants (*J*) are given in Hz. High resolution mass spectra were obtained either on an oa-TOF mass spectrometer with direct insertion and electron impact ionization (70 eV) or on an Ion Trap FT-MS using electrospray ionization in positive mode or APCI ionization in negative mode.

### Source of Enzymes

12-Oxophytodienoate reductase isoenzymes OPR1 and OPR3 from *Lycopersicon esculentum* and the OYE homologue YqjM from *Bacillus subtilis* were overexpressed and purified as reported.[17–19] The cloning, purification and characterization of OYE isoenzymes from yeast (OYE1 from *Saccharomyces pastorianus*, OYE2 and OYE3 from *Saccharomyces cerevisiae*) and nicotinamide-dependent cyclohexenone reductase (NCR) from *Zymomonas mobilis* were performed according to literature methods.[20, 1] Xenobiotic reductase XenA from *Pseudomonas putida*, and estrogen binding protein EBP1 from *Candida albicans* were obtained as published.[22–24] The cloning and characterization of GOYE from *Geobacillus kaustophilus* DSM 7263[25] and of CrS[26, 27] were performed as reported. The construction of *Lycopersicon esculentum* OPR1 variants Cys20Tyr, Ile287His, and His245Asp was recently published.[6]

### General Procedure for Bioreduction

An aliquot of enzyme (OPR1-wt, OPR3, OYE1–3, XenA, YqjM, EBP1, NCR, GkOYE, CrS, OPR1-C20Y, OPR1-I287H and OPR1-H245D; protein concentration in biotransformations 100 μg mL^−1^) was added to a 1.5-mL microcentrifuge tube containing Tris-HCl buffer solution (0.8 mL, 100 mM, pH 7.5), followed by the substrate (10 mM) and the cofactor (NADH, 15 mM). The mixture was shaken at 30 °C and 120 rpm. After 24 h the reaction was quenched by addition of aqueous HCl (4 N, 25 μL), products were extracted with ethyl acetate (0.8 mL). Methanol (100 μL) was added to the organic phase, and TMS-diazomethane solution (5 μL, 2 M in diethyl ether, obtained from Sigma Aldrich) were added for the derivatization of acids **2a** and **2b** to the corresponding methyl esters for GC analysis. After 30 min of incubation at room temperature, Na_2_SO_4_ was added and after filtration the conversion and *ee* of methyl esters of **2a** and **2b** were determined on GC. Control experiments without addition of enzymes were performed regularly.

### General Procedure for Deuterium Labelling Studies

Tris-HCl buffer (50 mM, pH 7.5) was prepared using D_2_O. To 800 μL of this solution, containing substrates (*E*)-**1a** methyl ester or (*E*)-**1b** (10 mM), NADH (15 mM), an aliquot of NCR and OPR1, respectively, were added (final protein concentration 200 μg mL^−1^). The mixture was shaken for 24 h at 30 °C and 120 rpm. This set-up was prepared ten-fold. The combined samples were extracted with CDCl_3_ (2×5 mL); the solution containing substrate (*E*)-**1b** was acidified to pH 1 using 4 M HCl prior to extraction. The phases were separated, denatured protein was removed by filtration and the organic solvent was evaporated. The residue was subjected to ^1^H NMR measurements using the following parameters: 16 scans and two dummy scans (48,000 data points); relaxation delay: 1 s; acquisition time 4 s; spectral width 6200 Hz; pulse sequence: 90° pulse, then acquisition.

### Supporting Information

Syntheses of (*E*)-**1a**, (*Z*)-**1a**, (*E*)-**1b**, (*Z*)-**1b**, *rac*-**2a**, (*R*)-**2b**, crude **5**, unlabelled and deuterium-labelled NMR spectra, analytical methods, optimization of the bioreduction and molecular modelling studies are available in the Supporting Information.

## Supporting Information

As a service to our authors and readers, this journal provides supporting information supplied by the authors. Such materials are peer reviewed and may be re-organized for online delivery, but are not copy-edited or typeset. Technical support issues arising from supporting information (other than missing files) should be addressed to the authors.

miscellaneous_information
