# Supplementary material for: Nitrile as Activating Group in the Asymmetric Bioreduction of β-Cyanoacrylic Acids Catalyzed by Ene-Reductases
Source: Adv Synth Catal. 2014 Apr 9;356(8):1878–82. doi: 10.1002/adsc.201301055 (PMC4498475; doi:10.1002/adsc.201301055)
Supplement: Supplementary file 1 — miscellaneous_information [file adsc0356-1878-sd1.pdf]

Advanced Synthesis & Catalysis

Supporting Information

© Copyright Wiley-VCH Verlag GmbH & Co. KGaA, 69451 Weinheim, 2014

Nitrile as Activating-Group in the Asymmetric Bioreduction of β-Cyano-Acrylic Acids Catalyzed by Ene-Reductases

Christoph K. Winkler,[a] Dorina Clay,[a] Nikolaus G. Turrini,[a] Horst Lechner,[a] Wolfgang Kroutil,[a] Simon Davies,[b] Sebastien Debarge,[b] Pat O'Neill,[b] Jeremy Steflik,[c] Mike Karmilowicz,[c] John W. Wong,[c],* Kurt Faber[a],*

a Department of Chemistry, Organic & Bioorganic Chemistry, University of Graz, Heinrichstrasse 28, A-8010 Graz, Austria; b Pfizer Global Supply, Process Development Centre, Loughbeg, County Cork, Ireland; c Pfizer Worldwide R&D, Chemical R&D, Eastern Point Rd, Groton, CT 06340, USA.

Supporting Information:

General procedure A for ester hydrolysis

A solution of ester (1.0 g) in MeOH (5 ml) was stirred with 0.49 g of aqueous NaOH (50% w/w) at 21°C. After 4 h, the reaction mixture was concentrated under reduced pressure and the remaining solid was suspended in isopropanol, filtered, and washed with isopropanol to give the products (*E*)- and (*Z*)-**1a** as the sodium salt.

(*E*)-3-Cyano-5-methylhex-2-enoic acid [(*E*)-**1a** sodium salt]: (*E*)-ethyl 3-cyano-5-methylhex-2-enoate[1] (1.0 g) was hydrolyzed according to general procedure A to give (*E*)-**1a** sodium salt as a white solid (0.36 g, 37%); ^{1}H NMR (400 MHz, D_2O) δ 6.62 (s, 1H), 2.16 (d, J = 7.2 Hz, 2H), 1.73 (m, 1H), 0.77 (d, J = 6.5 Hz, 6H); ^{13}C NMR (100 MHz, D_2O) δ 172.1, 142.2, 120.2, 116.8, 38.4, 27.0, 21.2; HRMS (ESI) calc for $C_8H_{10}NNa_2O_2^+$, 198.0501, found 198.0506.

(*Z*)-3-Cyano-5-methylhex-2-enoic acid [(*Z*)-**1a** sodium salt]: (*Z*)-ethyl 3-cyano-5-methylhex-2-enoate[1] (1.0 g) was hydrolyzed according to general procedure A to give (*Z*)-**1a** sodium salt as a white solid (0.50 g, 52%); ^{1}H NMR (400 MHz, D_2O) δ 6.37 (s, 1H), 2.08 (d, J = 7.1 Hz, 2H), 1.76 (m, 1H), 0.78 (d, J = 6.7 Hz, 6H); ^{13}C NMR (100 MHz, D_2O) δ 171.4, 142.6, 119.6, 118.6, 42.9, 26.8, 21.1; HRMS (ESI) calc for $C_8H_{10}NNa_2O_2^+$, 198.0501, found 198.0504.

(*E*)-3-Cyano-5-methylhexa-2,4-dienoic acid [(*E*)-**1b**]: Isobutyraldehyde (18.6 g, 0.258 mol) was added to a solution of cyanoacetic acid (20.0 g, 0.235 mol) in pyridine (200 ml). Pyrrolidine (4.0 g,

* Corresponding author information: (J.W.W.) Tel: +1-860-441-6882; E-mail: john.w.wong@pfizer.com. (K.F.) Tel: +43-316-380-5332; E-mail: kurt.faber@uni-graz.at.

0.056 mol) was then added and the resultant yellow solution was stirred overnight at room temperature. 50% Glyoxylic acid solution (34.8 g, 0.235 mol) was added and the solution stirred for 4.5 days at ambient temperature. The solution was poured into a mixture of conc. HCl (240 ml), water (400 ml) and toluene (200 ml). The toluene layer was separated, washed with warm water (150 ml) and combined with a toluene back-wash (100 ml) of the original aqueous quench solution. The toluene solution was concentrated to approximately 70 ml and stored overnight at -18°C. The product was filtered and washed with toluene and dried to afford (*E*)-**1b** (7.8 g, 22%) as a white crystalline solid; ^{1}H NMR (500 MHz, $CDCl_3$) δ 1.99 (3H, s), 2.15 (3H, s), 6.23 (1H, s), 6.85 (1H, s); ^{13}C NMR (125 MHz; $CDCl_3$): 19.9, 28.6, 117.1, 117.8, 127.0, 127.2, 150.9, 169.3; HRMS (ES+) *m/z* calcd for $C_8H_{10}NO_2$ $[M + H]^+$ 152.0712, found: 152.0708.

(*Z*)-3-Cyano-5-methylhexa-2,4-dienoic acid [(*Z*)-**1b**]

*2-Cyano-4-methylpent-2-enoic acid (**3**):* Isobutyraldehyde (9.3 g, 0.129 mol) was added to a solution of cyanoacetic acid (10.0 g, 0.118 mol) in pyridine (50 ml). Pyrrolidine (2.0 g, 0.028 mol) was added and the reaction stirred for 1 h. The reaction mixture was poured into a mixture of conc. HCl (60 ml) and H_2O (100 ml) and extracted with EtOAc (2 x 100ml). The combined organic layers were dried over anhydrous $MgSO_4$, filtered and concentrated *in vacuo* to give crude **3** as a white solid (15.30 g, 94%).

*3-Cyano-2-hydroxy-5-methylhex-3-enoic acid (**5**):* Crude 2-cyano-4-methylpent-2-enoic acid (**3**, 15.3 g) was dissolved in DMSO (150 mL) at ambient temperature and treated sequentially with DABCO (2.4 g, 20 mol%) and 50% glyoxylic acid (16.3 g, 0.11 mol). The solution was stirred at ambient temperature for 2 days and poured into a biphasic mixture of H_2O (300 mL) containing conc. HCl (10 mL) and CH_2Cl_2 (200 mL). The organic phase was separated, washed with H_2O and concentrated to afford crude **5** (13.4 g, 72%) as a mixture of diastereomers: ^{1}H NMR (500 MHz, D_2O) δ 1.70 (1.5 H, s, one diastereoisomer), 1.73 (1.5 H, s, one diastereoisomer), 1.75 (1.5 H, s, one diastereoisomer), 1.78 (1.5 H, s, one diastereoisomer), 3.89-3.98 (1 H, m, two diastereoisomers), 4.27 (0.5 H, d, one diastereoisomer), 4.50 (0.5 H, d, one diastereoisomer), 5.20-5.32 (1H, m, two diastereoisomers).

(*Z*)-**1b** potassium salt: Crude 3-cyano-2-hydroxy-5-methylhex-3-enoic acid (**5**, 13.4 g) was dissolved in MeOH (200 mL) and treated with K_2CO_3 (27.3 g). The suspension was stirred at ambient temperature overnight and filtered. Concentration of the filtrate gave (*Z*)-**1b** potassium salt as a white crystalline solid (15 g, 100%). ^{1}H NMR (500 MHz, D_2O) δ 1.87 (3H, s), 1.98 (3H, s), 5.83 (1H, s), 6.50 (1H, s); ^{13}C NMR (125 MHz; D_2O) δ 18.6, 26.7, 115.9, 117.9, 118.3, 141.4, 144.9, 171.7. HRMS (ES+) *m/z* calcd for $C_8H_{10}NO_2$ $[M + H]^+$ 152.0712, found: 152.0705.

Synthesis of racemic and nonracemic reference material

(*R*)-3-Cyano-5-methylhex-4-enoic acid [(*R*)-**2b**)] was prepared by bioreduction of (*E*)-**1b** using OPR1wt in a 150 ml reaction at 30°C containing (*E*)-**1b** (1.13 g, 7.5 mmol), 2-propanol (3.54 g, 59 mmol), *Lactobacillus brevis* alcohol dehydrogenase (4800 U), $NADP^+$ (115 mg, 0.15 mmol), and OPR1wt (9 g wet *E. coli* cells; constructed in pET28b and expressed in BL21 *E. coli* cells) and potassium phosphate buffer (100 mM, pH 7, 2 mM $MgCl_2$). After 22 h, the reaction was extracted with EtOAc. The EtOAc extract was dried over anhydrous $MgSO_4$, concentrated under reduced pressure and eluted through silica gel (5 g) to give 1.15 g of (*R*)-**2b** as a light orange oil (95.6%, >99 % ee determined by GC analysis on Chiraldex™ G-TA 30 m x 0.25 mm id, 135°C isothermal); $[\alpha]^{20}_D = -83.6$ (c 1.13, MeOH); ^{1}H NMR (400 MHz, $CDCl_3$) δ 9.8 (br. s, 1H), 5.07 – 5.03 (m, 1H), 3.78 (dd, *J* = 16.2, 7.2 Hz, 1H), 2.79 (dd, *J* = 16.9, 7.4 Hz, 1H), 2.61 (dd, *J* = 16.9, 7.0 Hz, 1H), 1.70 (d, *J* = 13.1 Hz, 6H); ^{13}C NMR (100 MHz, $CDCl_3$) δ 175.2, 139.5, 119.8, 116.7, 37.3, 25.78, 25.47, 18.2; HRMS (ES^+): m/z calcd. for $C_8H_{15}N_2O_2$ $[M+NH_4]^+$ 171.1128, found 171.1127.

The absolute configuration of the bioreduction product (*R*)-**2b** was confirmed by partial hydrogenation of (*R*)-**2b** (200 mg, 1.3 mmol) with Pd on C (200 mg, 5%) in 60 ml of isopropanol at 30 °C. After 4 h, the reaction was filtered and concentrated under reduced pressure to give (*S*)-**2a** (163 mg, 80%) with >99% ee (determined by GC analysis on Chiraldex G-TA 30 m x 0.25 mm id) 135°C isothermal). Spectroscopic data for (*S*)-**2a** were consistent with data for *rac*-**2a**.

rac-3-Cyano-5-methylhexanoic acid (*rac*-**2a** sodium salt): *rac*-ethyl 3-cyano-5-methylhexanoate[1] was hydrolyzed according to general procedure A to give *rac*-**2a** sodium salt as a white solid (0.63 g, 55%); ^{1}H NMR (400 MHz, CD_3OD) δ 3.16 – 3.08 (m, 1H), 2.51 (dd, *J* = 15.6, 7.8 Hz, 1H), 2.40 (dd, *J* = 15.6, 6.8 Hz, 1H), 1.88 – 1.79 (m, 1H), 1.63 – 1.56 (ddd, *J* = 13.6, 10.9, 5.0 Hz, 1H), 1.46 - 1.40 (ddd, *J* = 13.6, 9.4, 5.0 Hz, 1H), 1.00 (dd, *J* = 6.4, 5.7 Hz, 6H); ^{13}C NMR (100 MHz, D_2O) δ 178.5, 124.2, 39.95, 39.85, 26.8, 25.7, 22.1, 20.7.

rac-3-Cyano-5-methylhex-4-enoic acid (*rac*-**2b**) was prepared by treating (*R*)-**2b** (100 mg) in 5 ml of sodium borate buffer (0.1 M, pH 10.6) at 60 °C for 12 h. The reaction mixture was then adjusted to pH 2 with 4 N HCl and extracted with EtOAc. The EtOAc extract was dried over anhydrous $MgSO_4$, filtered, and concentrated under reduced pressure to give *rac*-**2b** as a viscous oil (100 mg, 100%); $[\alpha]^{20}_D = -1.7$ (c 1.0, MeOH); 7.4% ee of (*R*)-**2b** was determined by GC analysis on Chiraldex™ G-TA 30 m x 0.25 mm id (135°C isothermal). Spectroscopic data for (*rac*)-**2b** were consistent with data for (*R*)-**2b**.

(*S*)-Methyl 3-cyano-5-methylhexanoate [(*S*)-**2a** methyl ester] was prepared according to literature.[1]

GC-analyses

Chiral and achiral GC analyses were carried out using a Hydrodex-β-TBDAc capillary column (25 m x 0.25 mm id). Injector temperature: 250 °C, flow rate: 0.8 mL/min. The method was 140 °C hold 10 min, 10 °C min^{-1} to 160 °C, hold 10 min. Chiral GC analyses were also carried out using a Chiraldex G-TA column (30 m x 0.25 mm id).

Table S1. Retention times on chiral GC.

Compound	Retention time [min]	
	(*E*)	(*Z*)
**1a methyl ester**	8.15	14.65
**1b methyl ester**	3.47	6.73
	(*R*)	(*S*)
**2a methyl ester**	8.99	9.15
**2b methyl ester**	6.11	5.96

Figure S1. Optimization of pH and buffer type of bioreduction.

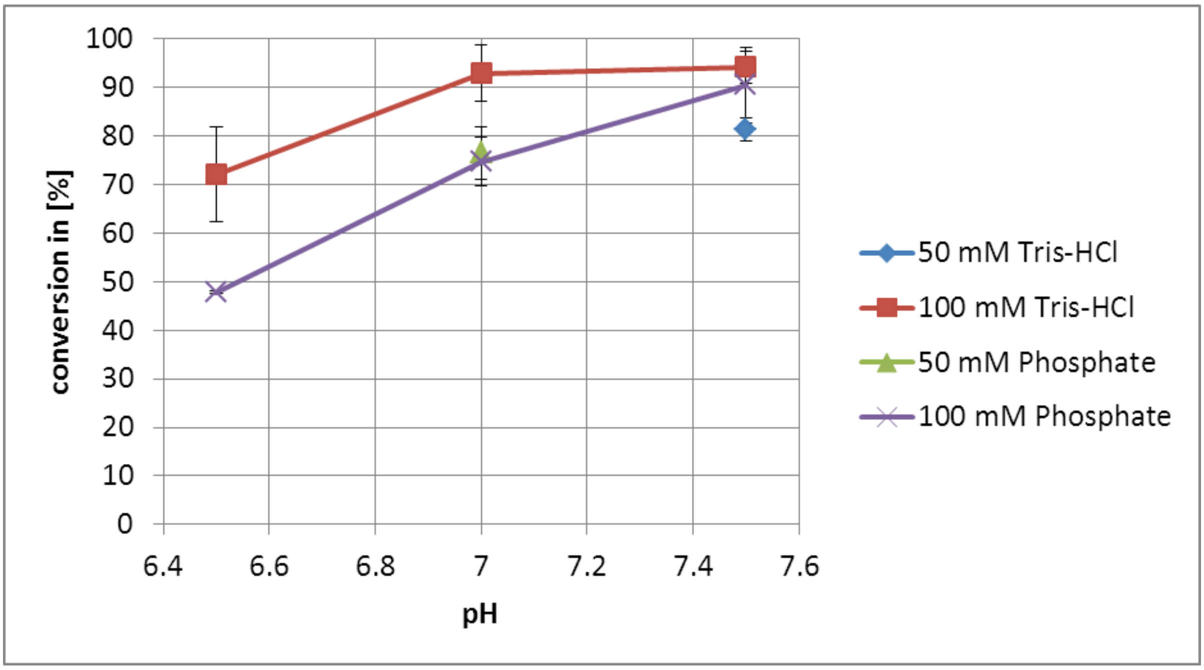

Molecular Modeling Studies

For docking studies, chain A of the structure of OPR1 (PDB code: 3HGR) was used as a monomer. All steps were carried out using the Schrödinger Maestro package (Maestro version: 9.3.023 MMshare version: 2.1.023). The protein structure was prepared after deleting all ligands and water molecules with Protein Preparation Wizard (PPW) and the missing residues in loop β6 were added

with Prime (Glu11, Asp12, Lys21, Lys279, Thr290, Lys351, Glu373). During H-bond assignment, catalytically important His187 and His190 were re-oriented manually to provide their correct conformation. The FMN cofactor was simulated in its reduced state using chemical drawing program implemented the in the package. Finally, a retrained minimization (OPLS 2005, RMSD 0.3A) was done by the PPW. Comparison with the only available structure of an ene-reductase containing a reduced $FMNH_2$ (2GQA) revealed a similar bent cofactor conformation. Ligands were created using the Maestro drawing program and were prepared with the program LigPrep included in the package, using the OPLS 2005 force field and a pH of 7±0.5. Afterwards, a Macromodel-conformational search was carried out and all the conformers with an OPLS 2005 energy below 5 kcal/mol were used for docking. The grid was generated using Glide and ligands were docked using extra precision mode. The best structure of (*E*)-3-cyano-5-methylhexa-2,4-dienoic acid [(*E*)-**2a**] with a docking score of -3.904 was selected. No binding mode with a flipped orientation of the activating group (i.e. hydrogen bonding of the carboxylate instead of the nitrile to His187 and His190) was obtained.

References

[1] C. K. Winkler, D. Clay, S. Davies, P. O'Neill, P. McDaid, S. Debarge, J. Steflik, M. Karmilowicz, J. W. Wong, K. Faber, *J. Org. Chem.* **2013**, *78*, 1525-1533.

^{1}H- and ^{13}C-NMR Spectra:

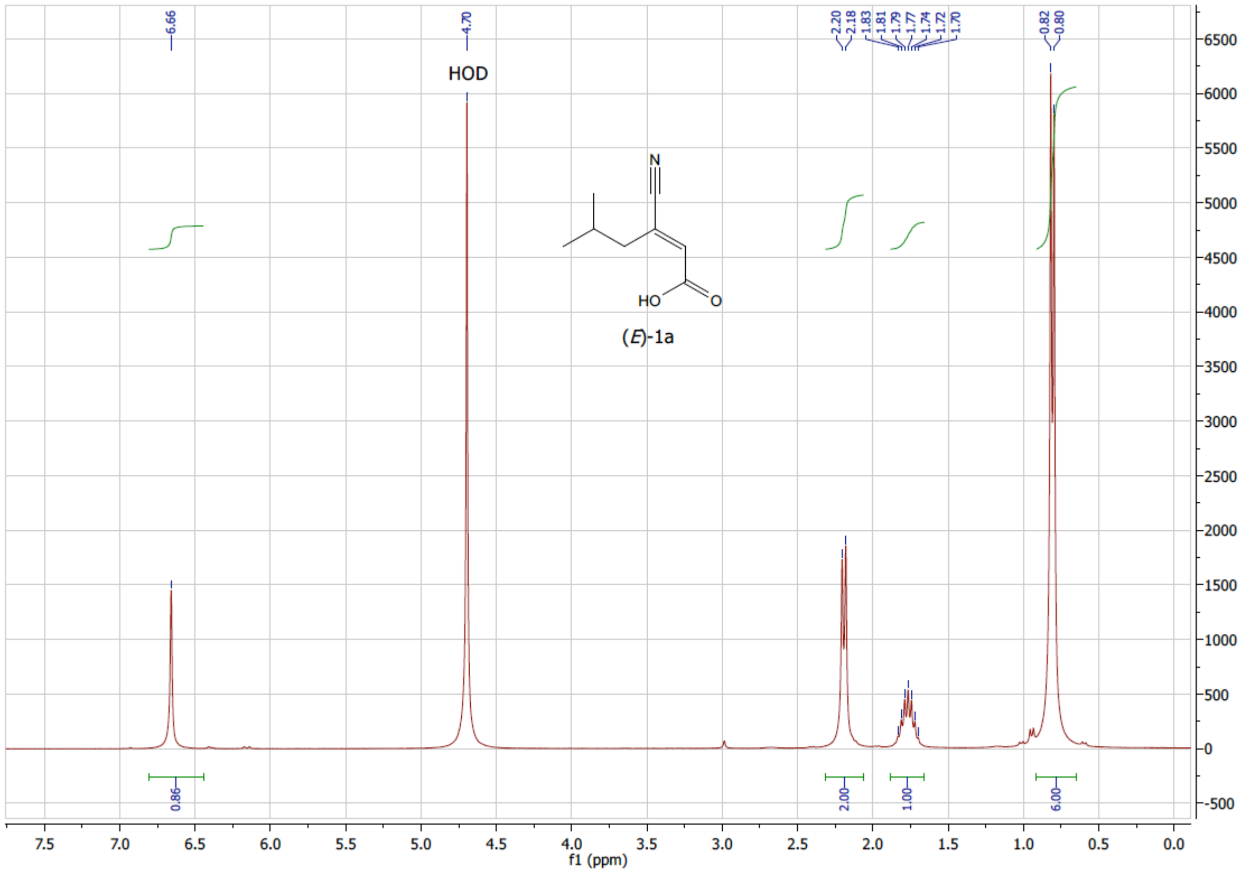

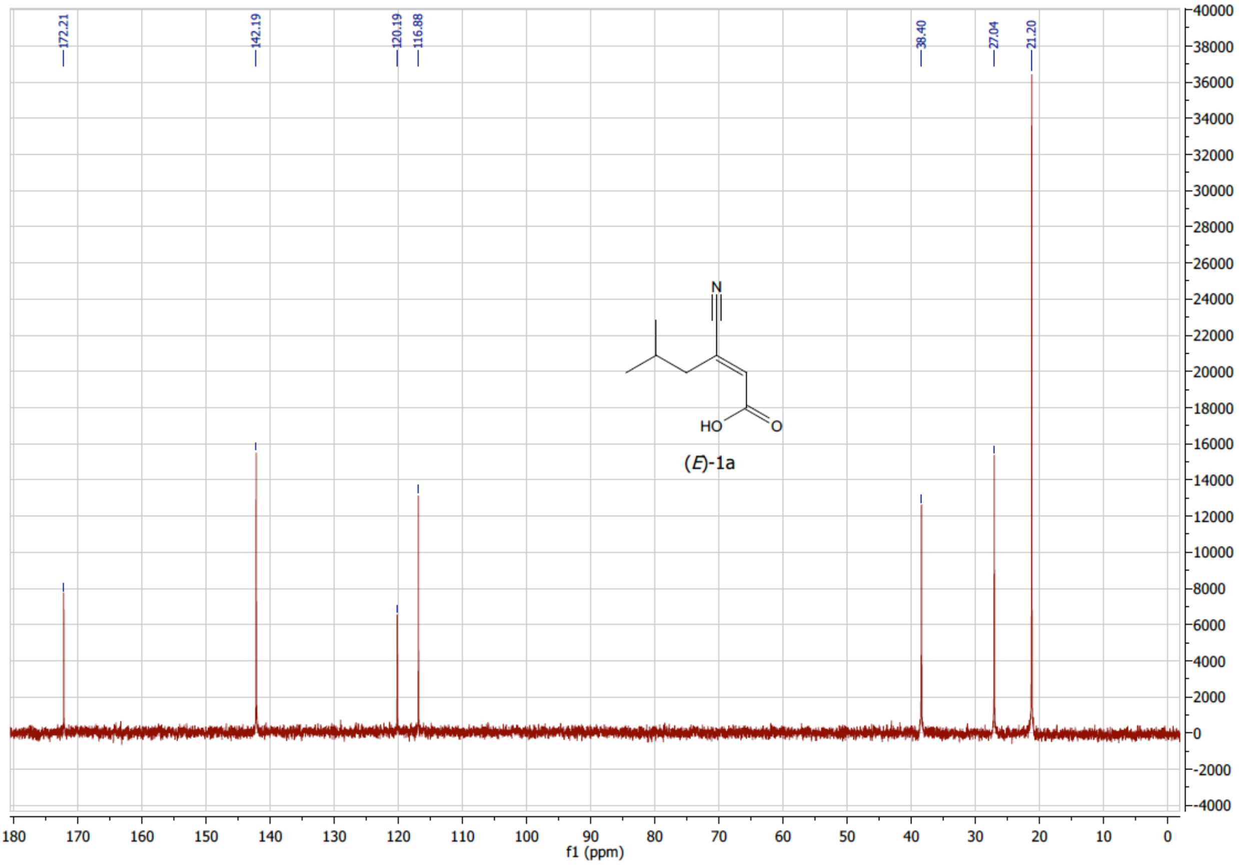

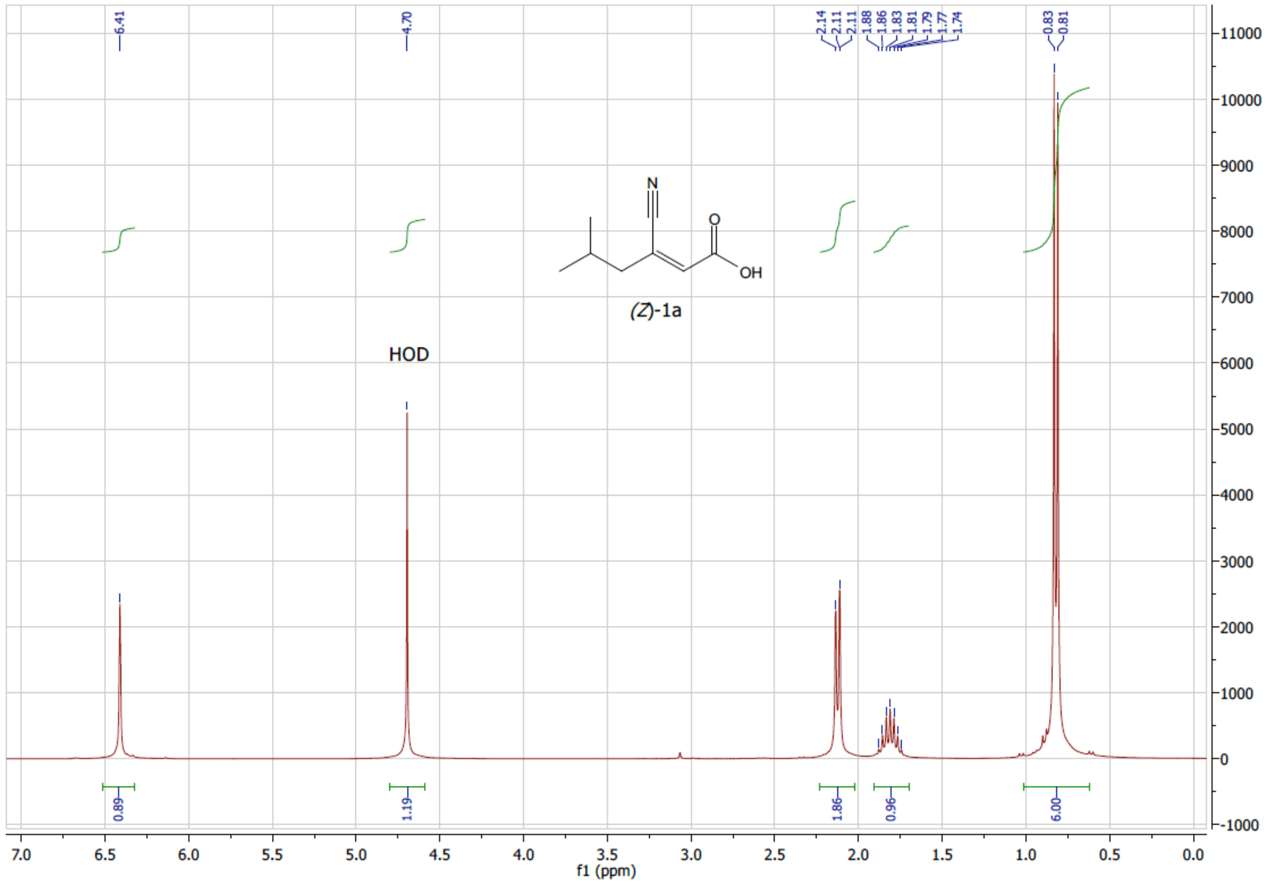
6.41
4.70
2.14
2.11
2.11
1.88
1.86
1.83
1.81
1.79
1.77
1.74
0.83
0.81
N
O
OH
(Z)-1a
HOD
0.89
1.19
1.86
0.96
6.00
f1 (ppm)

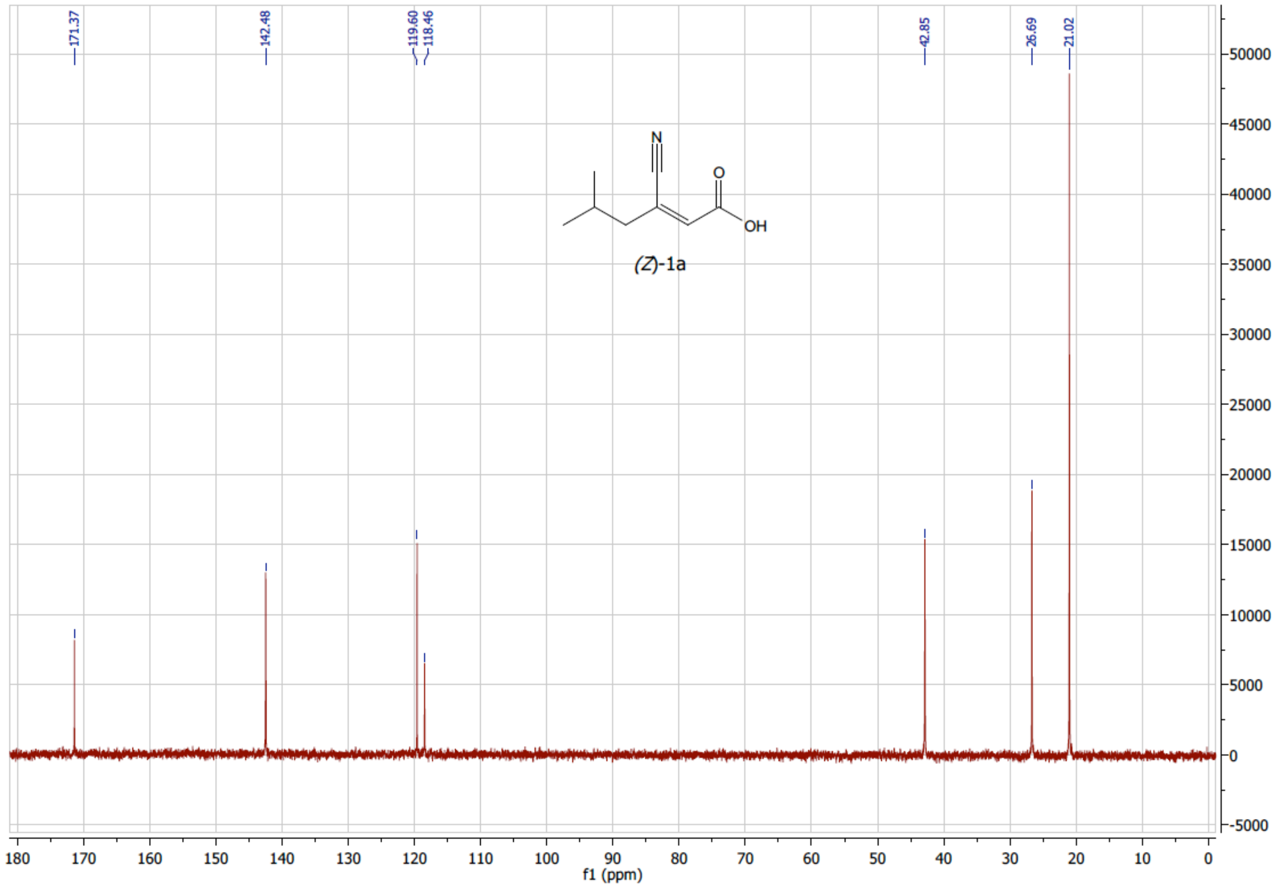
171.37
142.48
119.60
118.46
42.85
26.69
21.02
N
O
OH
(Z)-1a
f1 (ppm)

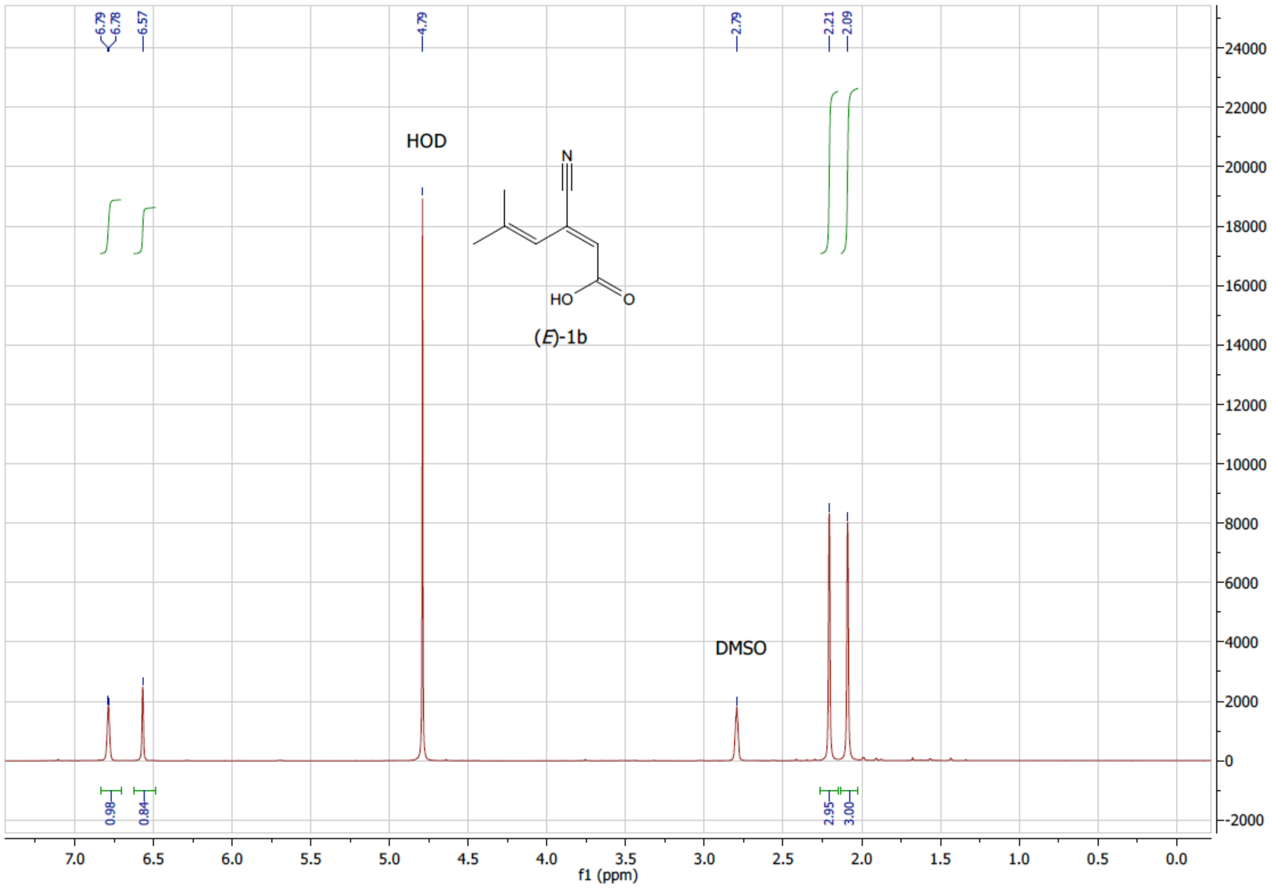
6.79
6.78
6.57
4.79
2.79
2.21
2.09
HOD
DMSO
(E)-1b
0.98
0.84
2.95
3.00
f1 (ppm)

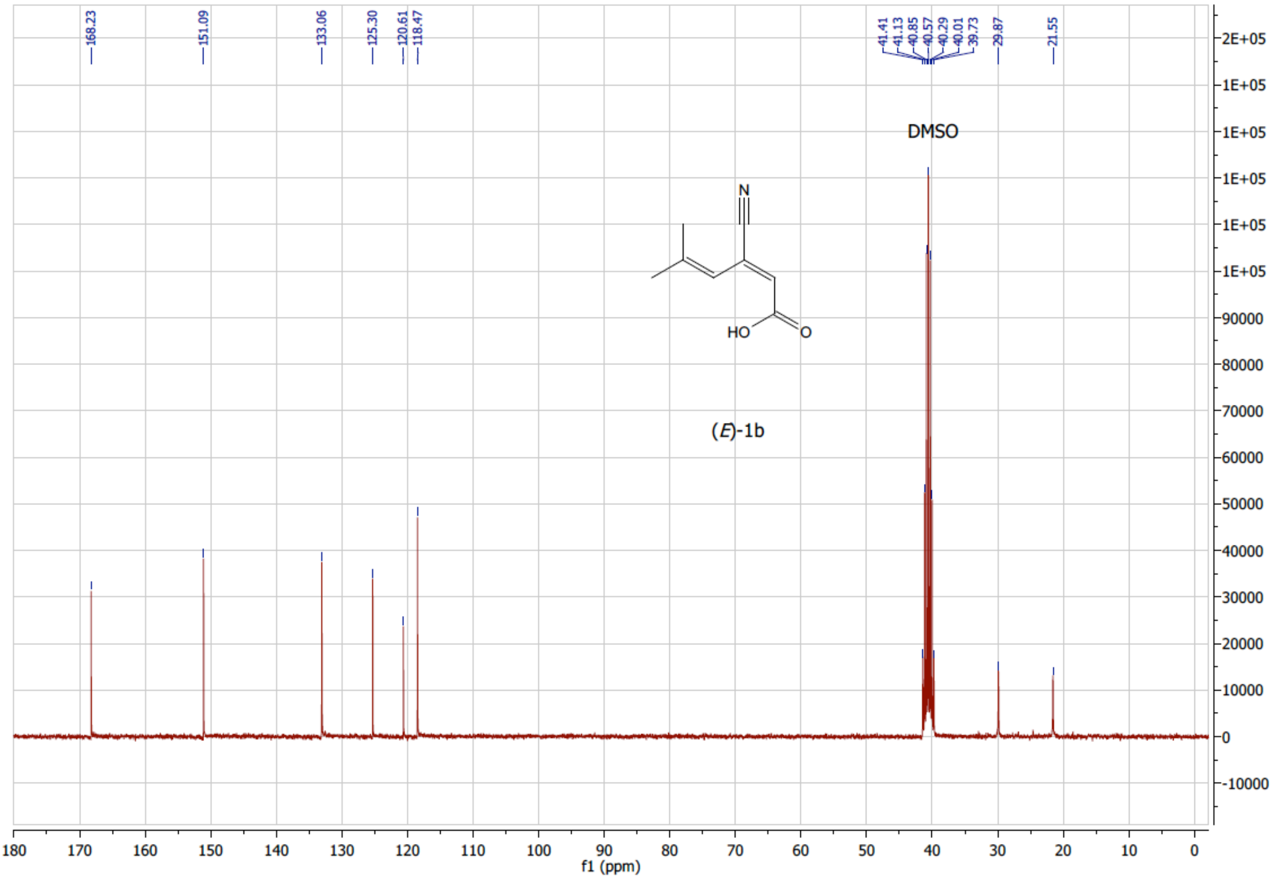
168.23
151.09
133.06
125.30
120.61
118.47
41.41
41.13
40.85
40.57
40.29
40.01
39.73
29.87
21.55
DMSO
(E)-1b
f1 (ppm)

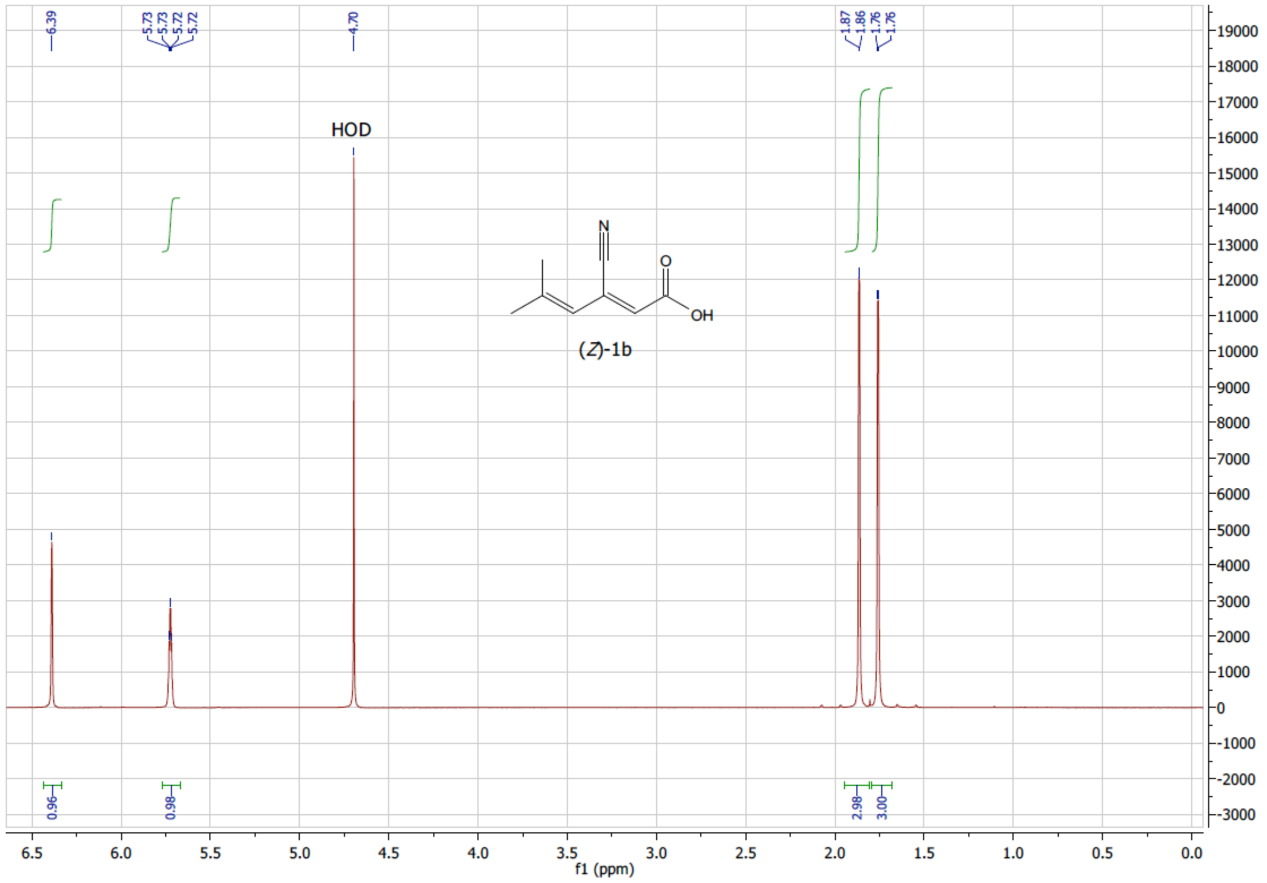

6.39
5.73
5.73
5.72
5.72
4.70
1.87
1.86
1.76
1.76
HOD
N
O
OH
(Z)-1b
0.96
0.98
2.98
3.00
f1 (ppm)

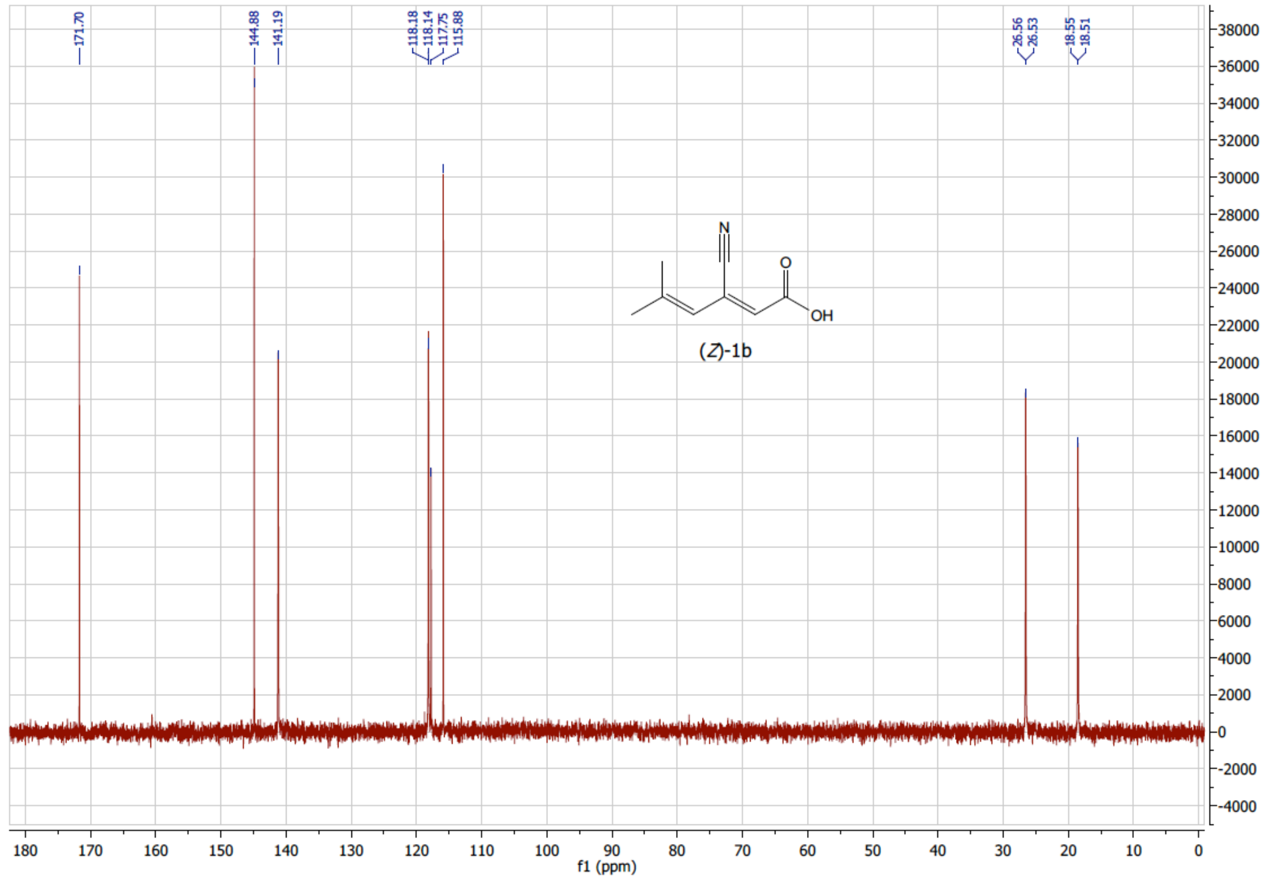

171.70
144.88
141.19
118.18
118.14
117.75
115.88
26.56
26.53
18.55
18.51
N
O
OH
(Z)-1b
f1 (ppm)

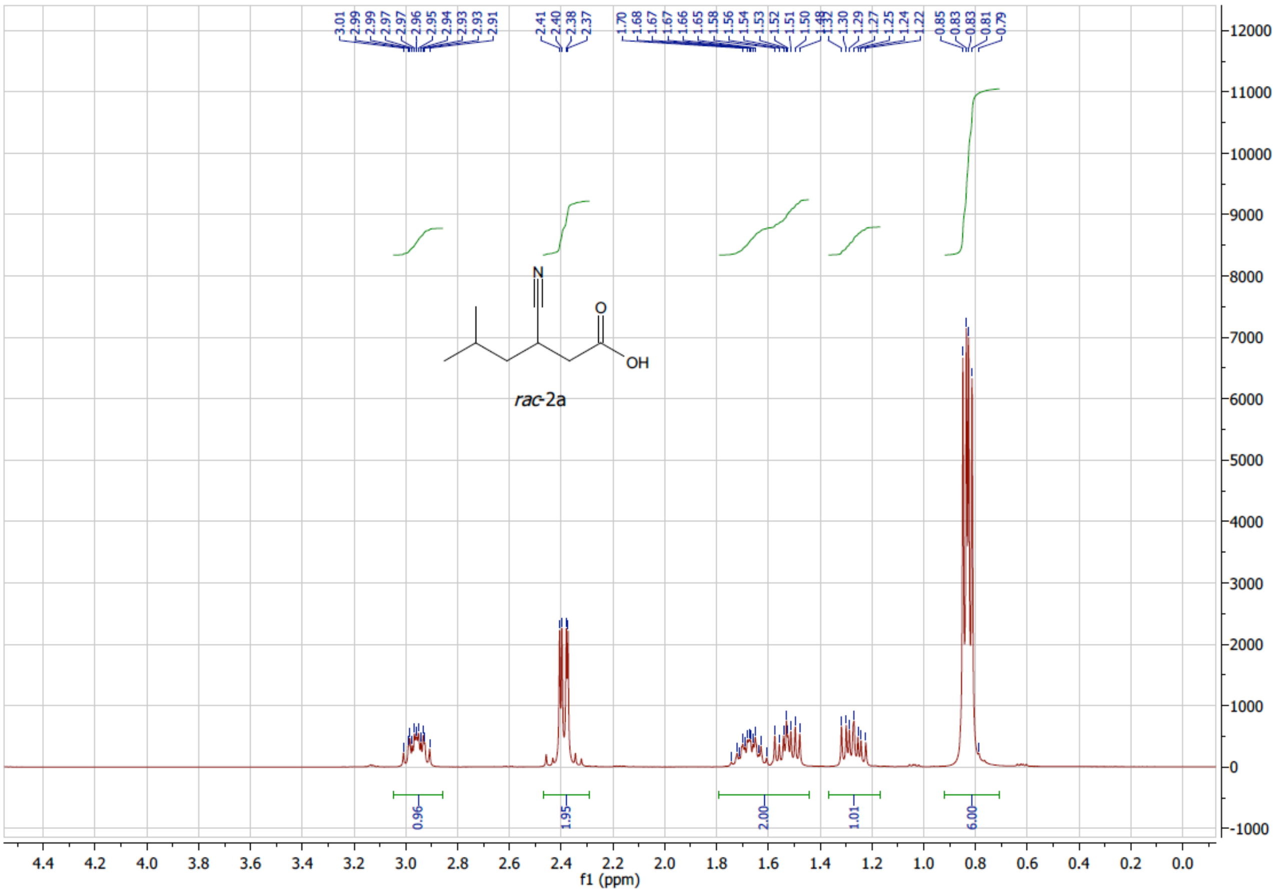
rac-2a
f1 (ppm)

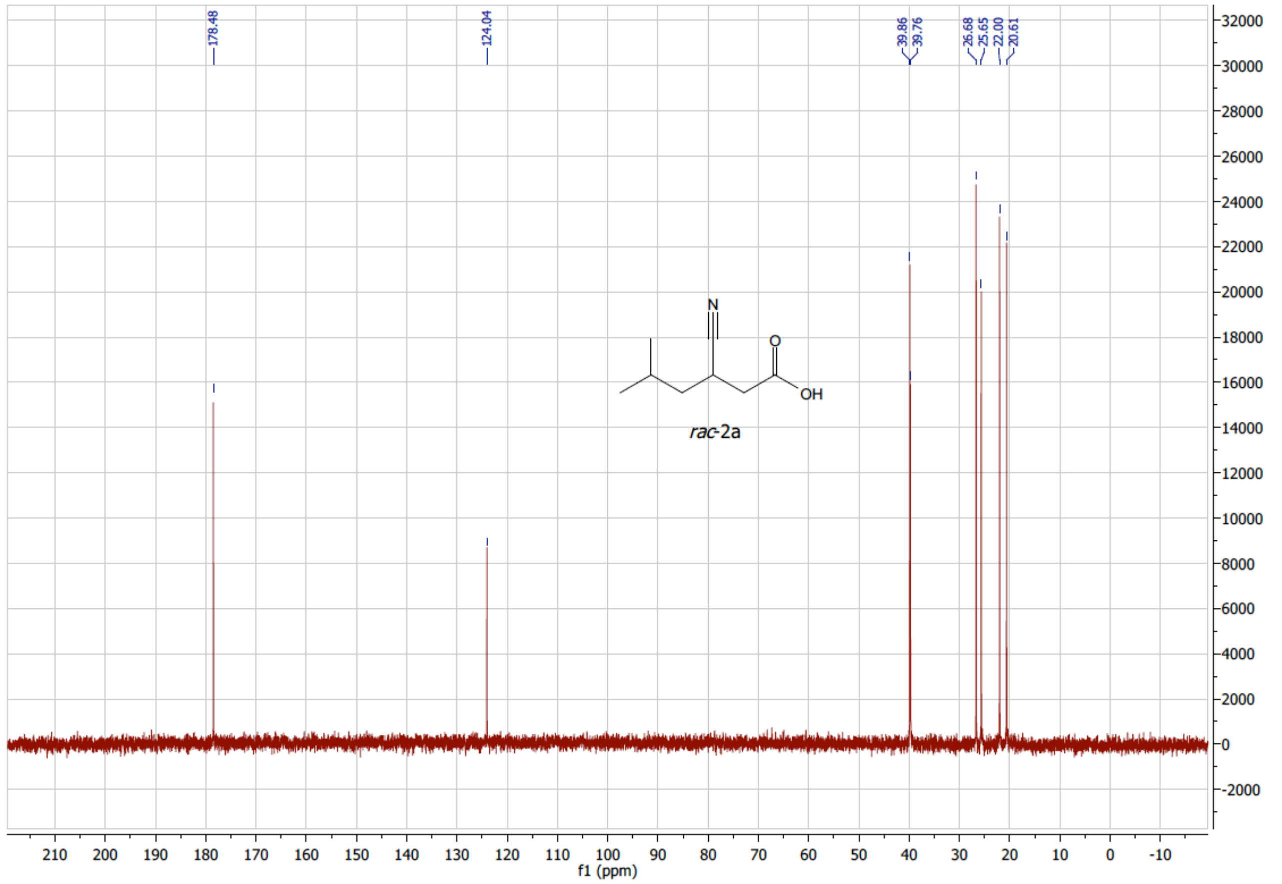
rac-2a
f1 (ppm)

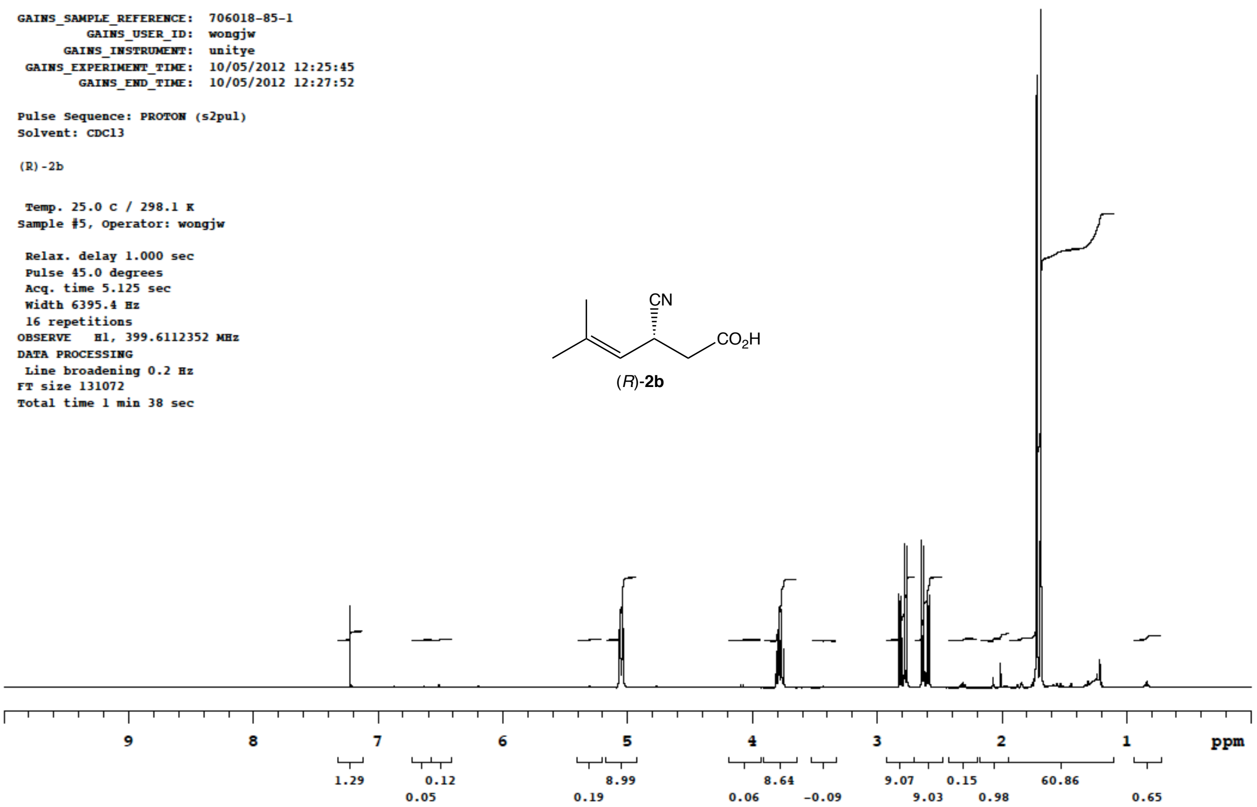
GAINS_SAMPLE_REFERENCE: 706018-85-1
GAINS_USER_ID: wongjw
GAINS_INSTRUMENT: unitye
GAINS_EXPERIMENT_TIME: 10/05/2012 12:25:45
GAINS_END_TIME: 10/05/2012 12:27:52
Pulse Sequence: PROTON (s2pul)
Solvent: CDCl3
(R)-2b
Temp. 25.0 C / 298.1 K
Sample #5, Operator: wongjw
Relax. delay 1.000 sec
Pulse 45.0 degrees
Acq. time 5.125 sec
Width 6395.4 Hz
16 repetitions
OBSERVE H1, 399.6112352 MHz
DATA PROCESSING
Line broadening 0.2 Hz
FT size 131072
Total time 1 min 38 sec
CN
CO_2H
(*R*)-**2b**
9 8 7 6 5 4 3 2 1 ppm
1.29 0.12 0.05 8.99 0.19 8.64 0.06 -0.09 9.07 9.03 0.15 0.98 60.86 0.65

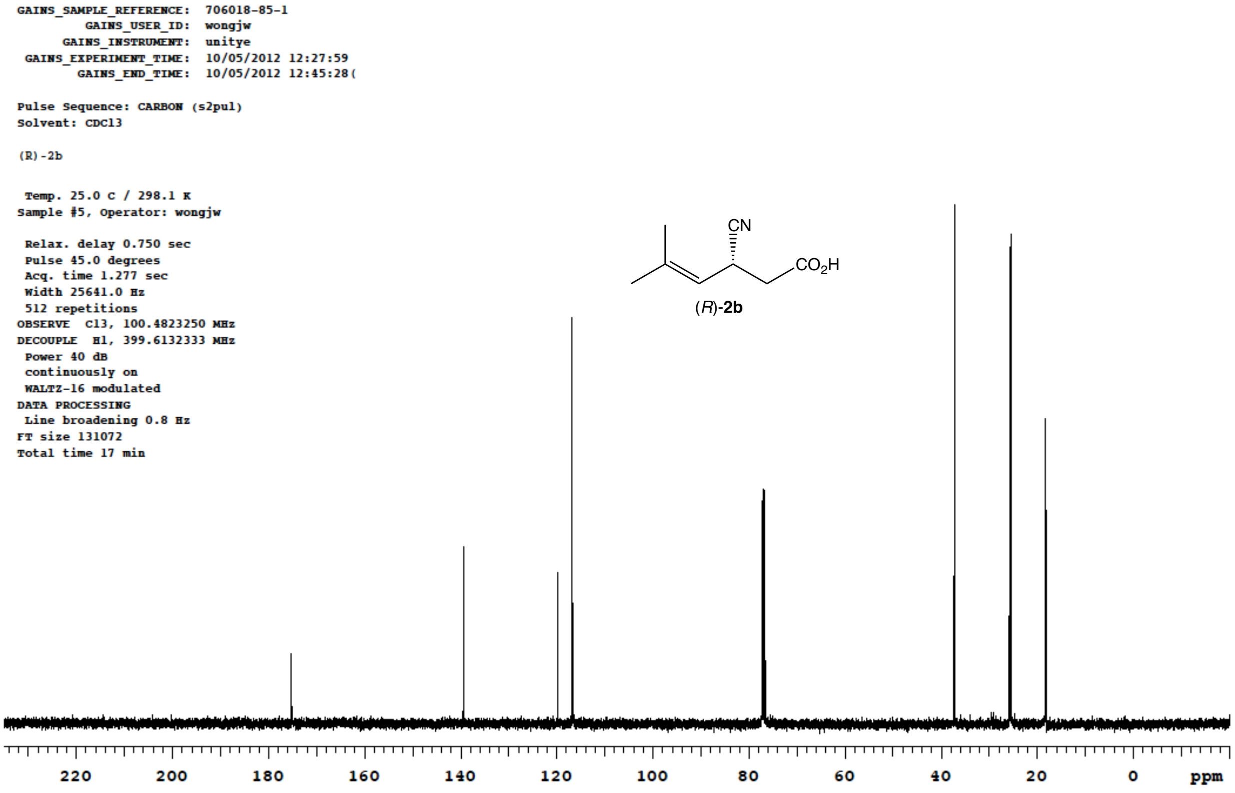
GAINS_SAMPLE_REFERENCE: 706018-85-1
GAINS_USER_ID: wongjw
GAINS_INSTRUMENT: unitye
GAINS_EXPERIMENT_TIME: 10/05/2012 12:27:59
GAINS_END_TIME: 10/05/2012 12:45:28(
Pulse Sequence: CARBON (s2pul)
Solvent: CDCl3
(R)-2b
Temp. 25.0 C / 298.1 K
Sample #5, Operator: wongjw
Relax. delay 0.750 sec
Pulse 45.0 degrees
Acq. time 1.277 sec
Width 25641.0 Hz
512 repetitions
OBSERVE C13, 100.4823250 MHz
DECOUPLE H1, 399.6132333 MHz
Power 40 dB
continuously on
WALTZ-16 modulated
DATA PROCESSING
Line broadening 0.8 Hz
FT size 131072
Total time 17 min
CN
CO_2H
(*R*)-**2b**
220 200 180 160 140 120 100 80 60 40 20 0 ppm

^{1}H-NMR Spectra from deuterium labelling:

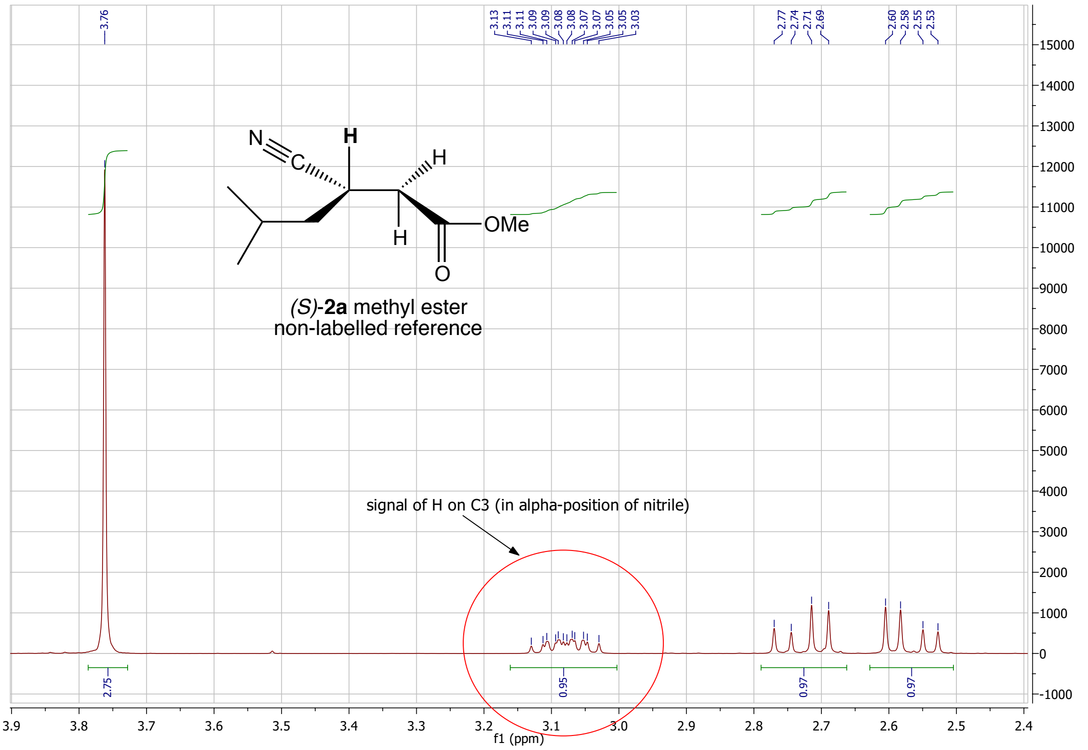

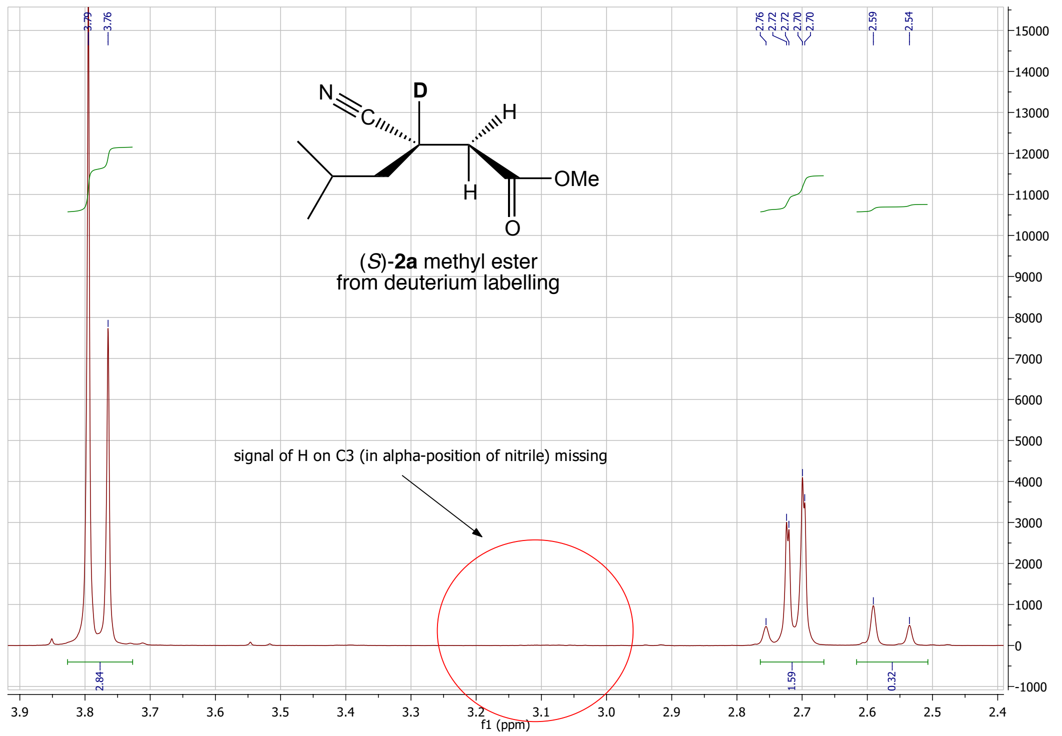

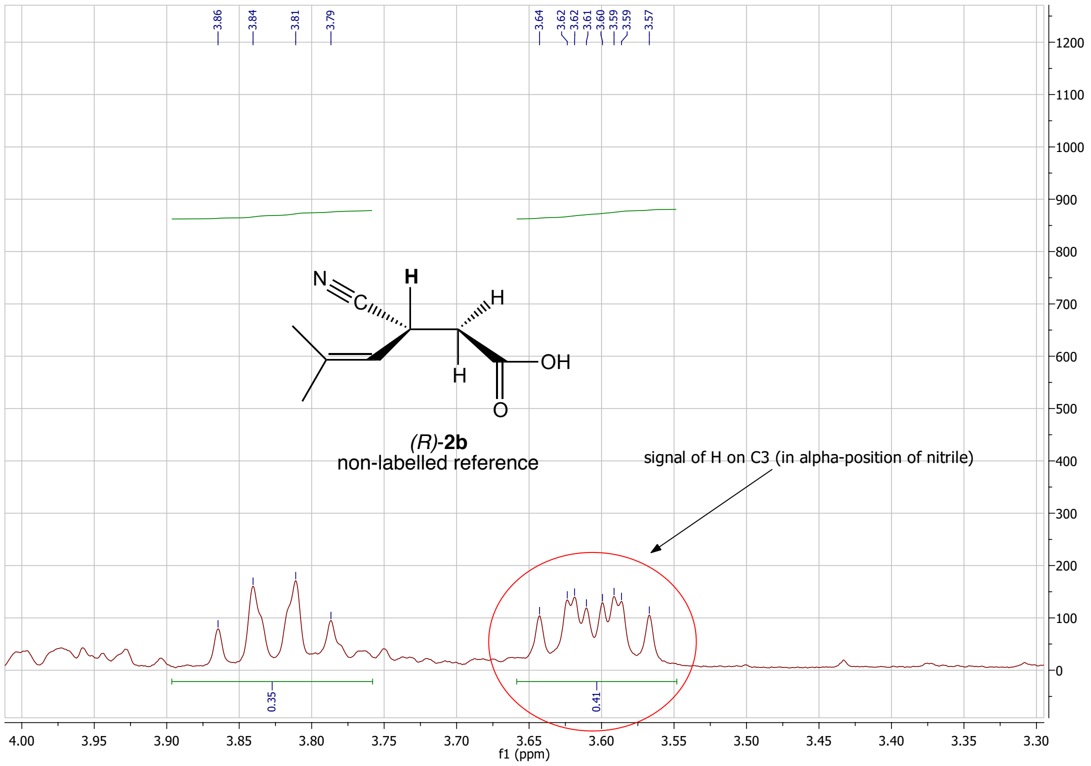
3.86
3.84
3.81
3.79
3.64
3.62
3.62
3.61
3.60
3.59
3.59
3.57
N
C
H
H
H
OH
O
(R)-2b
non-labelled reference
signal of H on C3 (in alpha-position of nitrile)
0.35
0.41
f1 (ppm)

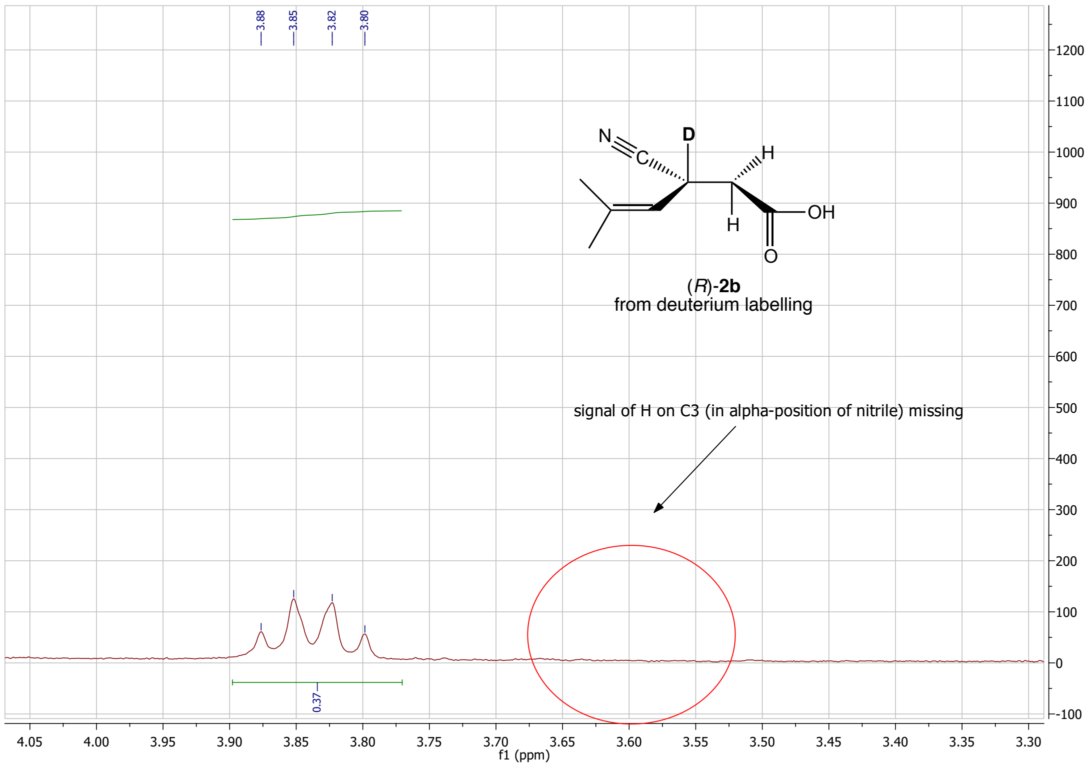
3.88
3.85
3.82
3.80
N
C
D
H
H
OH
O
(R)-2b
from deuterium labelling
signal of H on C3 (in alpha-position of nitrile) missing
0.37
f1 (ppm)

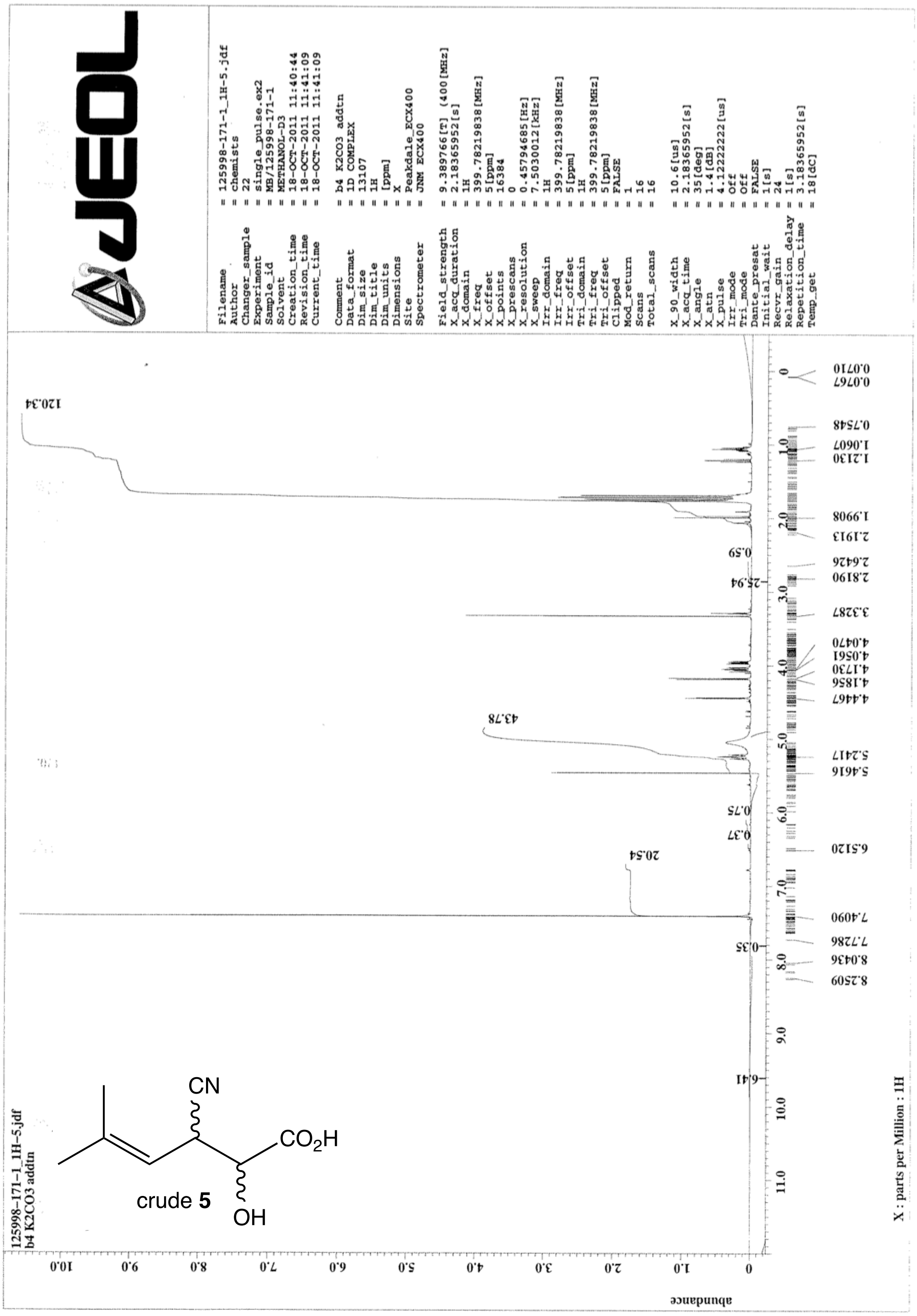

125998-171-1_1H-5.jdf
b4 K2CO3 addtn
CN
CO2H
OH
crude 5
JEOL
Filename = 125998-171-1_1H-5.jdf
Author = chemists
Changer_sample = 22
Experiment = single_pulse.ex2
Sample_id = MB/125998-171-1
Solvent = METHANOL-D3
Creation_time = 18-OCT-2011 11:40:44
Revision_time = 18-OCT-2011 11:41:09
Current_time = 18-OCT-2011 11:41:09
Comment = b4 K2CO3 addtn
Data_format = 1D COMPLEX
Dim_size = 13107
Dim_title = 1H
Dim_units = [ppm]
Dimensions = X
Site = Peakdale_ECX400
Spectrometer = JNM ECX400
Field_strength = 9.389766[T] (400[MHz]
X_acq_duration = 2.18365952[s]
X_domain = 1H
X_freq = 399.78219838[MHz]
X_offset = 5[ppm]
X_points = 16384
X_prescans = 0
X_resolution = 0.45794685[Hz]
X_sweep = 7.5030012[kHz]
Irr_domain = 1H
Irr_freq = 399.78219838[MHz]
Irr_offset = 5[ppm]
Tri_domain = 1H
Tri_freq = 399.78219838[MHz]
Tri_offset = 5[ppm]
Clipped = FALSE
Mod_return = 1
Scans = 16
Total_scans = 16
X_90_width = 10.6[us]
X_acq_time = 2.18365952[s]
X_angle = 35[deg]
X_atn = 1.4[dB]
X_pulse = 4.12222222[us]
Irr_mode = Off
Tri_mode = Off
Dante_presat = FALSE
Initial_wait = 1[s]
Recvr_gain = 24
Relaxation_delay = 1[s]
Repetition_time = 3.18365952[s]
Temp_get = 18[dC]
120.34
43.78
20.54
6.41
0.35
0.37
0.75
25.94
0.59
8.2509
8.0436
7.7286
7.4090
6.5120
5.4616
5.2417
4.4467
4.1856
4.1730
4.0561
4.0470
3.3287
2.8190
2.6426
2.1913
1.9908
1.2130
1.0607
0.7548
0.0767
0.0710
abundance
X : parts per Million : 1H
